# Multivitamin supplementation as a potential adjunctive therapy in post-cardiac arrest: insights from a multicenter retrospective analysis of MIMIC-IV and eICU-CRD

**DOI:** 10.3389/fnut.2025.1602372

**Published:** 2025-09-23

**Authors:** Jiahao Shen, Danjun Wang, Wenxuan Zhao, Jun Que, Junwei Qian, Xiaoyun Zhang

**Affiliations:** ^1^Trauma Center, Tongji Hospital, School of Medicine, Tongji University, Shanghai, China; ^2^Department of Anesthesiology, Shanghai Geriatric Medical Center, Shanghai, China; ^3^Tianjin Medical University, Tianjin, China; ^4^Department of Neurosurgery, Jiangnan University Medical Center, Jiangnan University, Wuxi, China; ^5^Department of Thoracic Surgery, First Affiliated Hospital with Nanjing Medical University, Nanjing, China; ^6^Department of Emergency Medicine, Huashan Hospital, Fudan University, Shanghai, China; ^7^Department of Rheumatology, Huashan Hospital, Fudan University, Shanghai, China

**Keywords:** multivitamin supplementation, post-cardiac arrest, survival outcomes, retrospective cohort study, propensity score matching, inverse probability treatment weighting

## Abstract

**Background:**

Cardiac arrest (CA) remains a global health challenge, with low survival rates despite advances in resuscitation. There is a need for novel therapies to improve post-resuscitation outcomes, and the potential role of multivitamin supplementation in this context remains underexplored.

**Methods:**

This multi-center, retrospective observational study examined the association between multivitamin supplementation and clinical outcomes in CA patients. Data were derived from two publicly available critical care databases: the Medical Information Mart for Intensive Care IV (MIMIC-IV) as the training cohort and the eICU Collaborative Research Database (eICU-CRD) as the validation cohort. Adult CA patients were identified and categorized based on whether they received multivitamin supplementation during hospitalization. The primary outcome was in-hospital mortality, with ICU and 28-day mortality as secondary outcomes. Kaplan–Meier survival analysis, propensity score matching (PSM), and inverse probability treatment weighting (IPTW) were used to adjust for confounding variables. A stepwise Cox proportional hazards model evaluated the association between multivitamin use and mortality. Subgroup analyses were conducted based on age, gender, disease severity scores, and comorbidities. Stratified analyses were also performed for patients hospitalized ≥5 days, comparing outcomes between those receiving multivitamins for <5 days vs. ≥5 days.

**Results:**

In the MIMIC-IV cohort, 223 patients received multivitamins and 890 did not; in the eICU-CRD cohort, 174 received multivitamins and 2,455 did not. Patients receiving multivitamin supplementation had significantly higher survival rates for both in-hospital and 28-day mortality (*p* < 0.01). After PSM and IPTW adjustment, multivitamin use remained significantly associated with lower mortality in both cohorts. In the fully adjusted Cox model, hazard ratios for in-hospital mortality were 0.56 (95% CIs: 0.45–0.70), 0.47 (95% CIs: 0.35–0.62), and 0.52 (95% CIs: 0.42–0.65) in the original, PSM, and IPTW analyses, respectively (all *p* < 0.01). Subgroup analysis showed stronger effects in patients aged <65 years. Prolonged supplementation (≥5 days) was linked to better survival.

**Conclusion:**

Across two large critical care cohorts, multivitamin supplementation was associated with lower mortality after adjustment, consistent with a potential adjunctive role in post-cardiac arrest care.

## Introduction

Cardiac arrest (CA) remains a major global health challenge, with low rates of return of spontaneous circulation and survival to hospital discharge despite advancements in resuscitation strategies (1, 2). The incidence of out-of-hospital cardiac arrest ranges from 30.0 to 97.1 per 100,000 population, with survival rates as low as 3.1–20.4% worldwide (3, 4). Following successful resuscitation, ischemia–reperfusion (I/R) injury exacerbates multi-organ dysfunction through excessive reactive oxygen species (ROS) production, mitochondrial dysfunction, and systemic inflammation (5). ROS exert cytotoxic and pro-inflammatory effects on endothelial and neuronal cells, amplifying cytokine production. Additionally, they react with nitric oxide to form peroxynitrite, a potent oxidant that promotes oxidative damage, atherosclerosis, and thrombogenesis (6). Considering the crucial role of reactive oxygen species ROS in I/R injury, targeting oxidative stress and inflammation may prove to be essential strategies for improving outcomes in patients following cardiac arrest.

Multivitamin supplementation has been widely studied for its potential health benefits, particularly in relation to oxidative stress and inflammation. A study on postoperative parenteral nutrition with multivitamins showed a reduction in oxidative stress and improved metabolic transition, highlighting the benefits of multivitamin supplementation in managing oxidative stress (7). Individual vitamins, such as vitamin B, C, and D, have demonstrated significant effects in reducing oxidative stress and improving outcomes in various disease models. For example, vitamin C has been shown to mitigate myocardial dysfunction and reduce ROS levels following resuscitation (8, 9). High-dose vitamin B supplementation enhances oxidative metabolism and reduces markers of oxidative stress and inflammation (10). Vitamin D, with its immune-regulatory and antioxidant properties, plays a role in modulating oxidative stress and inflammation, which has been associated with improved cardiovascular outcomes (11, 12). However, the majority of studies have primarily examined the effects of individual vitamins, with limited research on the combined effects of multivitamins, particularly in critically ill patients, including those following cardiac arrest. To address this gap in research, we aim to investigate the potential impact of multivitamin supplementation on clinical outcomes in post-cardiac arrest patient.

This study aims to explore the relationship between multivitamin supplementation and clinical outcomes in CA patients through a multicenter retrospective analysis, using MIMIC-IV as the training cohort and eICU-CRD as the validation cohort. The findings may provide valuable insights into potential therapeutic strategies for improving clinical prognosis in CA patients.

## Materials and methods

### Study design and populations

This study is a multi-cohort, retrospective, observational study based on data from the Medical Information Mart for Intensive Care IV (MIMIC-IV) v3.0 database and the eICU Collaborative Research Database (eICU-CRD) v2.0 available at PhysioNet (certification number: 60311526). Patients with CA were identified in the MIMIC-IV database using ICD-9 code 427.5 and ICD-10 codes I46, I46.2, I46.8, and I46.9. In the eICU-CRD, CA cases were identified using ICD-9 code 427.5 and ICD-10 code I46.9, with diagnoses classified as either primary or major. Patients were excluded if they met any of the following criteria: (1) repeated ICU admissions; (2) age <18 years; (3) length of ICU stay <24 h; and (4) missing race information. Multivitamin use was identified from medication records: in MIMIC-IV and eICU we selected non-prenatal products whose name contained ‘multivitamin’ (case-insensitive). The exact selection logic, drug codes (‘ndc’ or ‘drughiclseqno’), and names are listed in Supplementary Table 1. Any oral, nasogastric, or intravenous administration during the index ICU stay classified a patient as a multivitamin user. This study was exempt from institutional review board (IRB) approval because the databases used fully de-identified data and had preexisting IRB approval. Patients from eICU-CRD were included as the discovery set while septic patients from MIMIC-IV were included in the validation set.

### Laboratory measurements and clinical characteristics

Clinical data were extracted from two databases using Structured Query Language (SQL) with PostgreSQL version 16.1. The variables collected included demographic characteristics (age, gender, race), scoring systems [Sequential Organ Failure Assessment (SOFA) score and Charlson Comorbidity Index (CCI)], laboratory parameters [hemoglobin, white blood cell (WBC) count, platelet count, international normalized ratio (INR), serum creatinine, blood glucose, bicarbonate, lactate, pH, and partial pressure of oxygen (pO_2_)], vital signs [heart rate (HR), respiratory rate (RR), mean arterial pressure (MAP), oxygen saturation (SpO_2_), and temperature (°C)], and ICU interventions (mechanical ventilation, vasopressor therapy, multivitamin supplementation, targeted temperature management (TTM), and renal replacement therapy (RRT)). Comorbidities were identified based on ICD-9 and ICD-10 codes.

### Outcomes

The primary outcome of interest was in-hospital mortality, while secondary outcomes included ICU mortality and 28-day mortality. Survival time was defined as the duration from ICU admission to either death or loss to follow-up at the end of the study, whichever occurred first. Patients discharged within 28 days or those surviving beyond 28 days were considered censored.

### Statistical analysis

The remaining missing data were imputed using multiple imputation with the “mice” package in R. Continuous variables following a normal distribution were expressed as mean ± standard deviation and compared using an independent samples t-test. For variables not conforming to a normal distribution, data were presented as median (interquartile range) and analyzed using the Mann–Whitney U test. Categorical variables were summarized as percentages and analyzed using Fisher’s exact test or the Chi-square test, as appropriate. Variables with more than 10% missing values were excluded from the analysis.

A stepwise Cox proportional hazards regression model was constructed to sequentially adjust for potential confounding factors and analyze the association between multivitamin supplementation and in-hospital mortality. The effect of multivitamin supplementation on patient outcomes was presented as hazard ratios (HRs) with 95% confidence intervals (CIs). Three models were constructed: Model I (adjusted for age, gender, and race), Model II (adjusted for age, gender, race, SOFA score, CCI score, hypertension, diabetes, myocardial infarction, congestive heart failure, chronic pulmonary disease, renal disease, and malignant cancer), and Model III (adjusted for age, gender, race, SOFA score, CCI score, hypertension, diabetes, myocardial infarction, congestive heart failure, chronic pulmonary disease, renal disease, malignant cancer, hemoglobin, WBC count, platelet count, INR, creatinine, blood glucose, bicarbonate, lactate, pH, pO_2_, heart rate, respiratory rate, SpO2, mechanical ventilation, vasopressor therapy, targeted temperature management, and renal replacement therapy).

Propensity score matching (PSM) analysis was conducted using a 1:1 nearest neighbor matching algorithm with a caliper of 0.02 to balance baseline characteristics between the two groups. In addition, inverse probability of treatment weighting (IPTW) was performed by calculating propensity scores through a logistic regression model, with weights assigned as the inverse probability of receiving or not receiving multivitamin supplementation. Standardized mean differences (SMDs) were calculated for all covariates, and kernel density plots were generated to assess balance. Subsequently, survival curves for in-hospital mortality and 28-day mortality were constructed for the matched groups, followed by stepwise Cox proportional hazards regression models to estimate HRs with sequential adjustments. Furthermore, subgroup analyses were performed to explore potential effect modifications based on age, gender, SOFA score, CCI score, and the presence of comorbidities, including hypertension, diabetes, myocardial infarction, congestive heart failure, chronic pulmonary disease, renal disease, and malignant cancer. Lastly, we performed a stratified analysis in patients hospitalized for >5 days, categorizing them based on whether multivitamin supplementation lasted for ≥5 days, to evaluate the impact of supplementation duration on survival outcomes.

All statistical analyses were conducted using R software (version 4.3.3), with a two-sided *p*-value <0.05 considered statistically significant.

## Results

### Baseline characteristics

**Figure 1:**
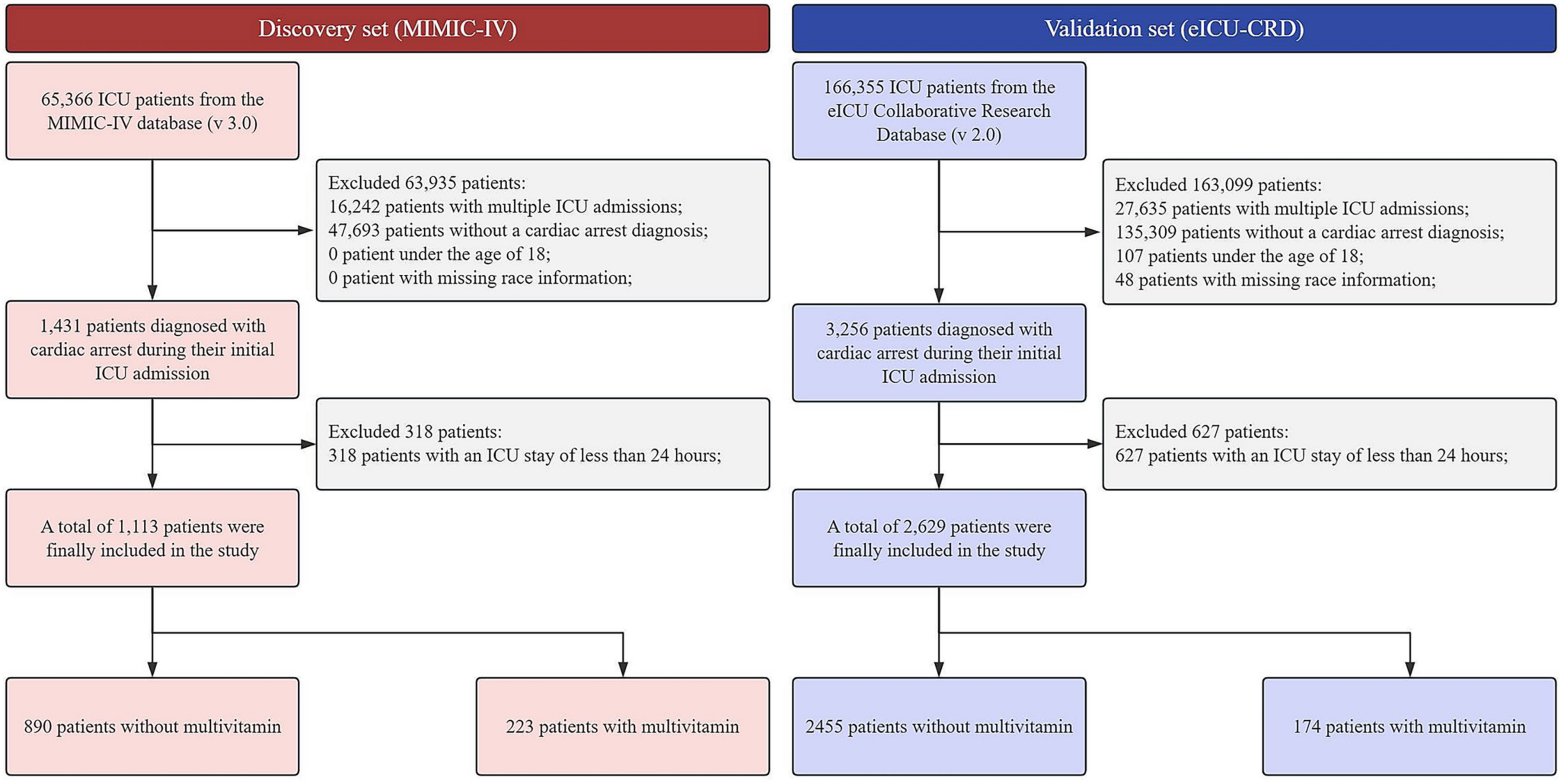
Patient selection flowchart for the MIMIC-IV and eICU-CRD cardiac arrest cohorts. ICU, intensive care unit; MIMIC-IV, medical information mart for intensive care IV; eICU-CRD, eICU collaborative research database.

Following the application of inclusion and exclusion criteria (Figure 1), the discovery cohort derived from the MIMIC-IV database included 1,113 patients, comprising 223 who received multivitamin supplementation and 890 who did not. Similarly, the validation cohort from the eICU-CRD comprised 2,629 patients, with 174 receiving multivitamin supplementation and 2,455 not receiving it.

The baseline characteristics of the MIMIC-IV cardiac arrest cohort are summarized in Table 1. The median age was 66 years (IQR: 55–78), and 62.3% were male. In terms of disease severity, the multivitamin group had a higher CCI score compared to the non-multivitamin group [6 (IQR: 4–8) vs. 5 (IQR: 3–8), *p* = 0.01]. Regarding specific comorbidities, the prevalence of myocardial infarction was similar between the multivitamin and non-multivitamin groups (29.1% vs. 30.6%, *p* = 0.74), while congestive heart failure was slightly higher in the multivitamin group (43.5% vs. 34.7%, *p* = 0.02). Laboratory parameters differed between the groups, with the multivitamin group exhibiting lower hemoglobin levels [11.1 g/dL (IQR: 9.0–12.9) vs. 11.8 g/dL (IQR: 9.8–13.7), *p* < 0.01], higher INR values [1.4 (IQR: 1.2–1.8) vs. 1.3 (IQR: 1.1–1.6), *p* = 0.01], and lower glucose levels [147 mg/dL (IQR: 109–208) vs. 165 mg/dL (IQR: 124–242), *p* < 0.01]. No significant differences were observed in the remaining laboratory parameters. Similarly, vital signs, including heart rate, respiratory rate, and oxygen saturation, showed no significant differences between the two groups. With regard to ICU interventions, patients in the multivitamin group were more likely to receive mechanical ventilation (87.9% vs. 82.5%, *p* = 0.06), vasopressors (80.7% vs. 70.3%, *p* < 0.01), targeted temperature management (43.0% vs. 44.2%, *p* = 0.82) and renal replacement therapy (22.4% vs. 10.9%, *p* < 0.01). Finally, outcome analysis revealed that ICU mortality was significantly lower in the multivitamin group compared to the non-multivitamin group (27.6% vs. 38.4%, *p* < 0.01). Similarly, in-hospital mortality was 32.8% vs. 46.2% (*p* < 0.01), and 28-day mortality was 93.1% vs. 97.6% (*p* < 0.01).

**Table 1:** Baseline characteristics of original patients in the MIMIC-IV cardiac arrest cohort.

Variables	All (*n* = 1,113)	Multivitamin (*n* = 223)	No multivitamin (*n* = 890)	*p-*value
Age, years	66 (55, 78)	67 (55, 77)	66 (54, 78)	0.97
Male, %	693 (62.3%)	150 (67.3%)	543 (61.0%)	0.10
CCI score	5 (4, 8)	6 (4, 8)	5 (3, 8)	0.01
SOFA score	7 (4, 10)	7 (5, 10)	7 (4, 10)	0.41
Race, *n* (%)				0.77
White	567 (50.9%)	116 (52%)	451 (50.7%)	
Black	106 (9.5%)	24 (10.8%)	82 (9.2%)	
Asian	22 (2%)	3 (1.3%)	19 (2.1%)	
Other	418 (37.6%)	80 (35.9%)	338 (38%)	
Comorbidities, *n* (%)				
Hypertension	418 (37.6%)	70 (31.4%)	348 (39.1%)	0.04
Diabetes mellitus	339 (30.5%)	69 (30.9%)	270 (30.3%)	0.93
Myocardial infarct	337 (30.3%)	65 (29.1%)	272 (30.6%)	0.74
Congestive heart failure	406 (36.5%)	97 (43.5%)	309 (34.7%)	0.02
Chronic pulmonary disease	270 (24.3%)	56 (25.1%)	214 (24.0%)	0.81
Renal disease	265 (23.8%)	64 (28.7%)	201 (22.6%)	0.07
Malignant cancer	114 (10.2%)	26 (11.7%)	88 (9.9%)	0.51
Laboratory tests				
Hemoglobin, g/dL	11.6 (9.6, 13.5)	11.1 (9.0, 12.9)	11.8 (9.8, 13.7)	<0.01
WBC, 10^9^/L	13.5 (9.5, 18.9)	12.4 (9.0, 18.1)	13.6 (9.6, 19.3)	0.09
Platelet, 10^9^/L	168 (125, 230)	161 (124, 224)	169 (125, 230)	0.53
INR	1.3 (1.1, 1.6)	1.4 (1.2, 1.8)	1.3 (1.1, 1.6)	0.01
Creatinine, mg/dl	1.2 (0.9, 1.9)	1.3 (0.9, 1.9)	1.2 (0.9, 1.9)	0.63
Glucose, mg/dl	162 (121, 238)	147 (109, 208)	165 (124, 242)	<0.01
Bicarbonate, mmol/L	21 (18, 24)	21 (18, 24)	21 (18, 24)	0.97
Lactate, mmol/L	2.6 (1.6, 5.0)	2.5 (1.6, 4.6)	2.6 (1.6, 5.0)	0.17
pH	7.3 (7.2, 7.4)	7.3 (7.2, 7.4)	7.3 (7.2, 7.4)	0.99
pO2, mmHg	94 (53, 196)	86 (47, 207)	98 (54, 189)	0.24
Vital signs				
HR, beats/min	81 (70, 95)	82 (71, 94)	80 (69, 95)	0.51
RR, times/min	20 (17, 23)	19 (17, 24)	20 (17, 23)	0.72
MBP, mmHg	77 (70, 85)	78 (70, 87)	77 (70, 85)	0.42
SpO2, %	97 (96, 99)	97 (96, 98)	97 (95, 99)	0.91
Temperature, °C	36.8 (36.5, 37.2)	36.8 (36.5, 37.1)	36.8 (36.4, 37.2)	0.60
Therapies, *n* (%)				
Mechanical ventilation	930 (83.6%)	196 (87.9%)	734 (82.5%)	0.06
Vasopressor	806 (72.4%)	180 (80.7%)	626 (70.3%)	<0.01
TTM	489 (43.9%)	96 (43.0%)	393 (44.2%)	0.82
RRT	147 (13.2%)	50 (22.4%)	97 (10.9%)	<0.01
Outcome				
ICU death	543 (48.8%)	92 (41.3%)	451 (50.7%)	0.01
In-Hospital death	613 (55.1%)	107 (48.0%)	506 (56.9%)	0.02
28-day death	630 (56.6%)	103 (46.2%)	527 (59.2%)	<0.01

CCI, Charlson comorbidity index; SOFA, sequential organ failure assessment; WBC, white blood cell count; INR, international normalized ratio; HR, heart rate; RR, respiratory rate; MBP, mean blood pressure; SpO_2_, peripheral capillary oxygen saturation; TTM, targeted temperature management; RRT, renal replacement therapy; ICU, intensive care unit.

In the validation cohort from the eICU-CRD (Supplementary Table 2), similar trends were observed in outcomes. ICU mortality, in-hospital mortality, and 28-day mortality were significantly lower in the multivitamin group compared to the non-multivitamin group (27.6% vs. 38.4%, *p* = 0.01; 32.8% vs. 46.2%, *p* < 0.01; and 90.2% vs. 97.0%, *p* < 0.01, respectively).

### Incrementally adjusted cox models assessing the impact of multivitamin supplementation on mortality

Supplementary Figure 1 illustrates the proportion of missing values for covariates, and covariates with more than 10% missing data were excluded from subsequent analyses. Table 2 presents the results of stepwise Cox regression models assessing the association between multivitamin supplementation and mortality outcomes in the MIMIC-IV cardiac arrest cohort.

**Table 2:** Hazard ratios for multivitamin use on mortality in MIMIC-IV cardiac arrest cohort.

Outcomes	Model 1		Model 2		Model 3	
	HR (95% CIs)	*p-*value	HR (95% CIs)	*p*-value	HR (95% CIs)	*p*-value
Original population						
ICU mortality						
Multivitamin	0.64 (0.51, 0.81)	<0.01	0.58 (0.47, 0.73)	<0.01	0.54 (0.42, 0.68)	<0.01
In-Hospital death						
Multivitamin	0.66 (0.54, 0.81)	<0.01	0.60 (0.49, 0.74)	<0.01	0.56 (0.45, 0.70)	<0.01
28-day death						
Multivitamin	0.61 (0.49, 0.75)	<0.01	0.56 (0.45, 0.69)	<0.01	0.53 (0.42, 0.66)	<0.01
After PSM						
ICU mortality						
Multivitamin	0.56 (0.43, 0.75)	<0.01	0.52 (0.40, 0.70)	<0.01	0.44 (0.33, 0.60)	<0.01
In-Hospital death						
Multivitamin	0.59 (0.46, 0.77)	<0.01	0.55 (0.42, 0.71)	<0.01	0.47 (0.35, 0.62)	<0.01
28-day death						
Multivitamin	0.56 (0.43, 0.73)	<0.01	0.53 (0.40, 0.69)	<0.01	0.46 (0.35, 0.60)	<0.01
After IPTW						
ICU mortality						
Multivitamin	0.54 (0.42, 0.70)	<0.01	0.51 (0.39, 0.66)	<0.01	0.50 (0.39, 0.63)	<0.01
In-Hospital death						
Multivitamin	0.57 (0.455, 0.72)	<0.01	0.53 (0.42, 0.68)	<0.01	0.52 (0.42, 0.65)	<0.01
28-day death						
Multivitamin	0.54 (0.43, 0.69)	<0.01	0.51 (0.40, 0.65)	<0.01	0.50 (0.40, 0.62)	<0.01

HR, hazard ratio; CIs, confidence intervals; ICU, intensive care unit; PSM, propensity score matching; IPTW, inverse probability of treatment weighting.

Model 1: Adjusted for demographic characteristics.

Model 2: Adjusted for demographic characteristics, disease severity, and comorbidities.

Model 3: Adjusted for demographic characteristics, disease severity, comorbidities, laboratory markers, and treatment interventions.

In the original population, Model 1, adjusted for demographic characteristics, showed that multivitamin supplementation was associated with a significant reduction in ICU mortality (HR: 0.64; 95% CI: 0.51–0.81; *p* < 0.01), in-hospital mortality (HR: 0.66; 95% CI: 0.54–0.81; *p* < 0.01), and 28-day mortality (HR: 0.61; 95% CI: 0.49–0.75; *p* < 0.01). After additional adjustment for disease severity and comorbidities in Model 2, the association remained robust, with HRs further reduced to 0.58 (95% CI: 0.47–0.73), 0.60 (95% CI: 0.49–0.74), and 0.56 (95% CI: 0.45–0.69) for ICU mortality, in-hospital mortality, and 28-day mortality, respectively (all *p* < 0.01). Model 3, the fully adjusted model, incorporated laboratory markers and ICU treatment interventions to further refine the association between multivitamin supplementation and mortality. Variance inflation factor (VIF) analysis, as shown in Supplementary Figure 2, demonstrated that all covariates had VIF values below 5, indicating no severe multicollinearity and ensuring the stability of the model estimates. After full adjustment, multivitamin supplementation remained significantly associated with lower mortality risk, with HRs of 0.54 (95% CI: 0.42–0.68) for ICU mortality, 0.56 (95% CI: 0.45–0.70) for in-hospital mortality, and 0.53 (95% CI: 0.42–0.66) for 28-day mortality (*p* < 0.01).

A similar trend was observed in the validation cohort from the eICU-CRD (Supplementary Table 3), where multivitamin supplementation was consistently associated with reduced ICU, in-hospital, and 28-day mortality in the original population. The association remained robust after sequential adjustments for disease severity, comorbidities, laboratory markers, and treatment interventions.

### Kaplan–Meier survival curves for in-hospital and 28-day mortality

**Figure 2:**
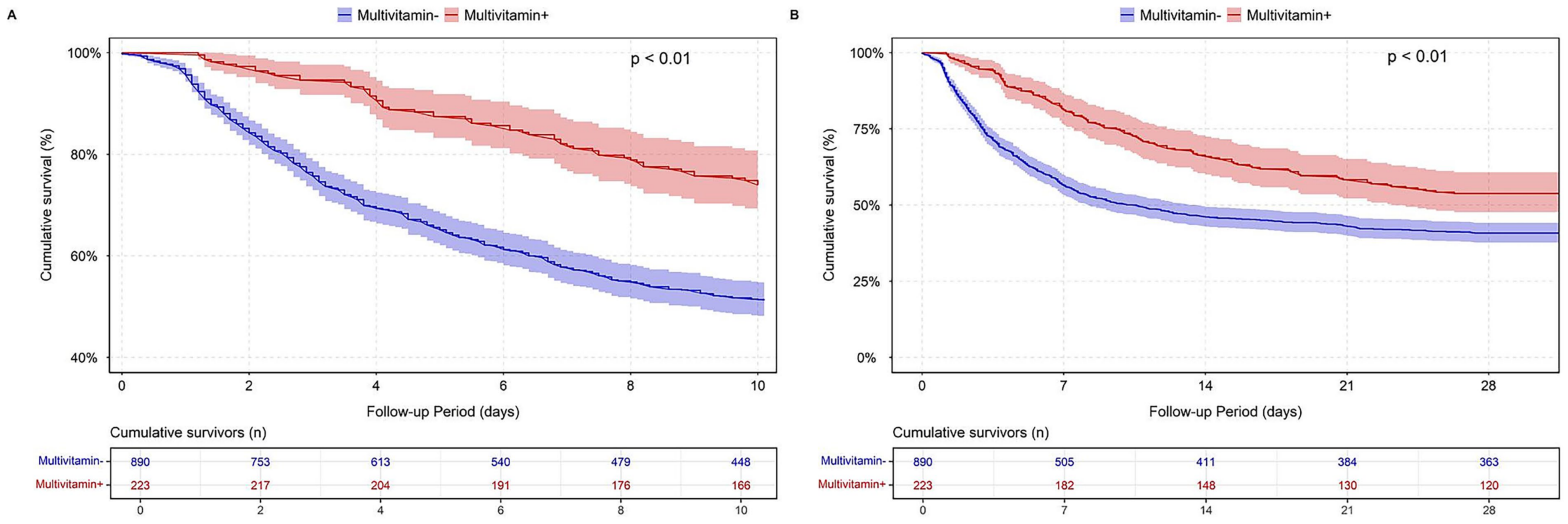
Survival analysis by multivitamin use in MIMIC-IV cardiac arrest cohort. **(A)** In-hospital survival; **(B)** 28-day survival.

In the MIMIC-IV cardiac arrest cohort, patients who received multivitamin supplementation exhibited a higher cumulative survival probability compared to those who did not. Log-rank tests indicated that the differences in both in-hospital and 28-day survival rates between the two groups were statistically significant (*p* < 0.01). The survival curves, as shown in Figure 2, illustrate the divergence in survival probability, with the multivitamin group maintaining a consistently higher survival rate throughout the follow-up period.

A similar trend was observed in the eICU-CRD cardiac arrest cohort, where multivitamin supplementation was associated with significantly higher in-hospital and 28-day survival (log-rank test, *p* < 0.01). The corresponding survival curves are shown in Supplementary Figure 3.

### Propensity score matching and inverse probability of treatment weighting in mortality analysis

To further minimize potential confounding, PSM and IPTW were performed to balance baseline characteristics between the groups in the MIMIC-IV cardiac arrest cohort. As illustrated in Supplementary Figure 4, SMD values for all covariates were reduced to below 0.1 after PSM and IPTW, indicating substantial improvement in covariate balance. Additionally, Supplementary Figure 5 presents kernel density plots of propensity score distributions, demonstrating improved overlap between groups after both matching and weighting.

Following PSM, 215 patients were included in each group. In the IPTW-adjusted analysis, the effective sample sizes were 869.72 in the control group and 171.89 in the multivitamin group, reflecting the weight-based adjustment of the cohort. The baseline characteristics of the PSM-matched population are summarized in Supplementary Table 4, with no statistically significant differences observed between the two groups across demographic characteristics, comorbidities, laboratory markers, or ICU interventions (*p* > 0.05). With respect to clinical outcomes, 28-day mortality remained significantly lower in the multivitamin group compared to the non-multivitamin group (46.0% vs. 60.9%, *p* < 0.01).

**Figure 3:**
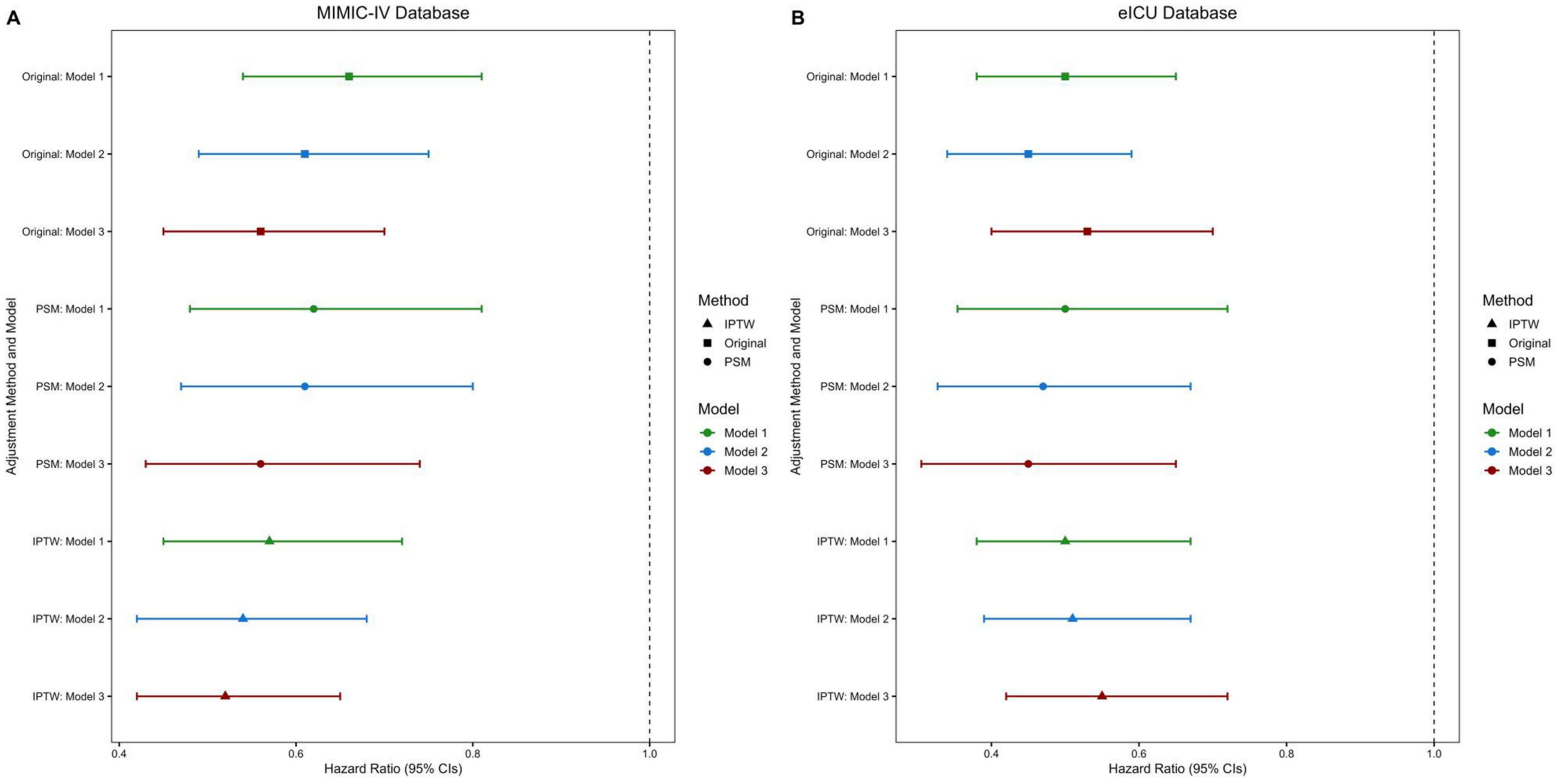
Forest plots of hazard ratios for multivitamin use on in-hospital mortality: **(A)** MIMIC-IV, **(B)** eICU-CRD. PSM, propensity score matching; IPTW, inverse probability of treatment weighting; HR, hazard ratio; CIs, confidence intervals. Analyses were performed in three populations: original, PSM, and IPTW, using three progressively adjusted Cox regression models. Model 1: Adjusted for demographic characteristics. Model 2: Adjusted for demographic characteristics, disease severity, and comorbidities. Model 3: Adjusted for demographic characteristics, disease severity, comorbidities, laboratory markers, and treatment interventions.

**Figure 4:**
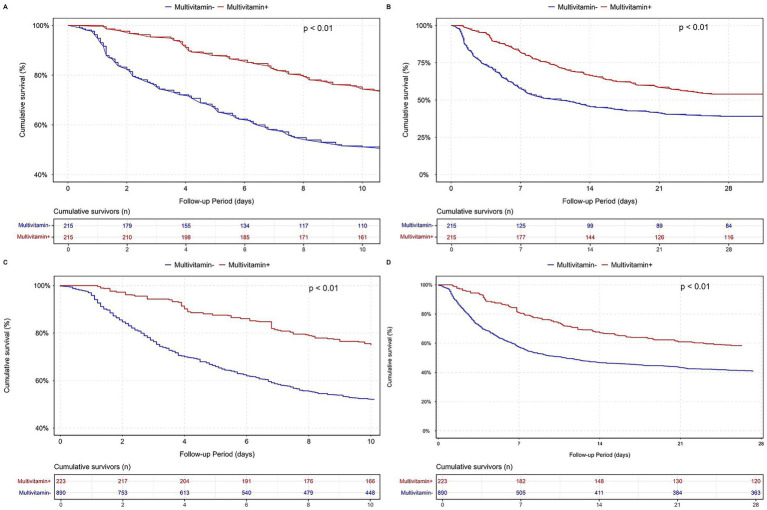
Survival analysis after PSM and IPTW for multivitamin use in the MIMIC-IV cardiac arrest cohort. Panel **(A)** shows the Kaplan–Meier survival curve for in-hospital mortality after PSM. Panel **(B)** displays the same comparison over a 28-day follow-up period after PSM. Panel **(C)** presents the in-hospital survival curve after IPTW, and panel **(D)** shows the 28-day survival curve after IPTW.

The PSM- and IPTW-adjusted analyses consistently demonstrated a robust association between multivitamin supplementation and reduced ICU, in-hospital, and 28-day mortality across the stepwise Cox regression models. In the fully adjusted model (Model 3), this protective effect persisted. Correspondingly, survival analyses further supported this association, as patients receiving multivitamin supplementation exhibited significantly higher cumulative survival probabilities than those who did not. As depicted in Figure 3, hazard ratios with 95% confidence intervals remained consistent across the original, PSM-matched, and IPTW-adjusted populations. Likewise, Figure 4 demonstrates a significant separation in in-hospital and 28-day survival curves between the two groups (log-rank test, *p* < 0.01), further supporting the observed survival advantage.

A similar trend was observed in the validation cohort of the eICU-CRD cardiac arrest population. PSM and IPTW effectively balanced baseline characteristics, as shown in the Supplementary material. After PSM, no significant differences remained between groups (Supplementary Table 5, *p* > 0.05). Stepwise Cox regression confirmed the association between multivitamin use and reduced mortality (Figure 3), while survival analyses demonstrated significantly higher cumulative survival in the multivitamin group (Supplementary Figure 6, log-rank test, *p* < 0.01).

### Subgroup analysis

**Figure 5:**
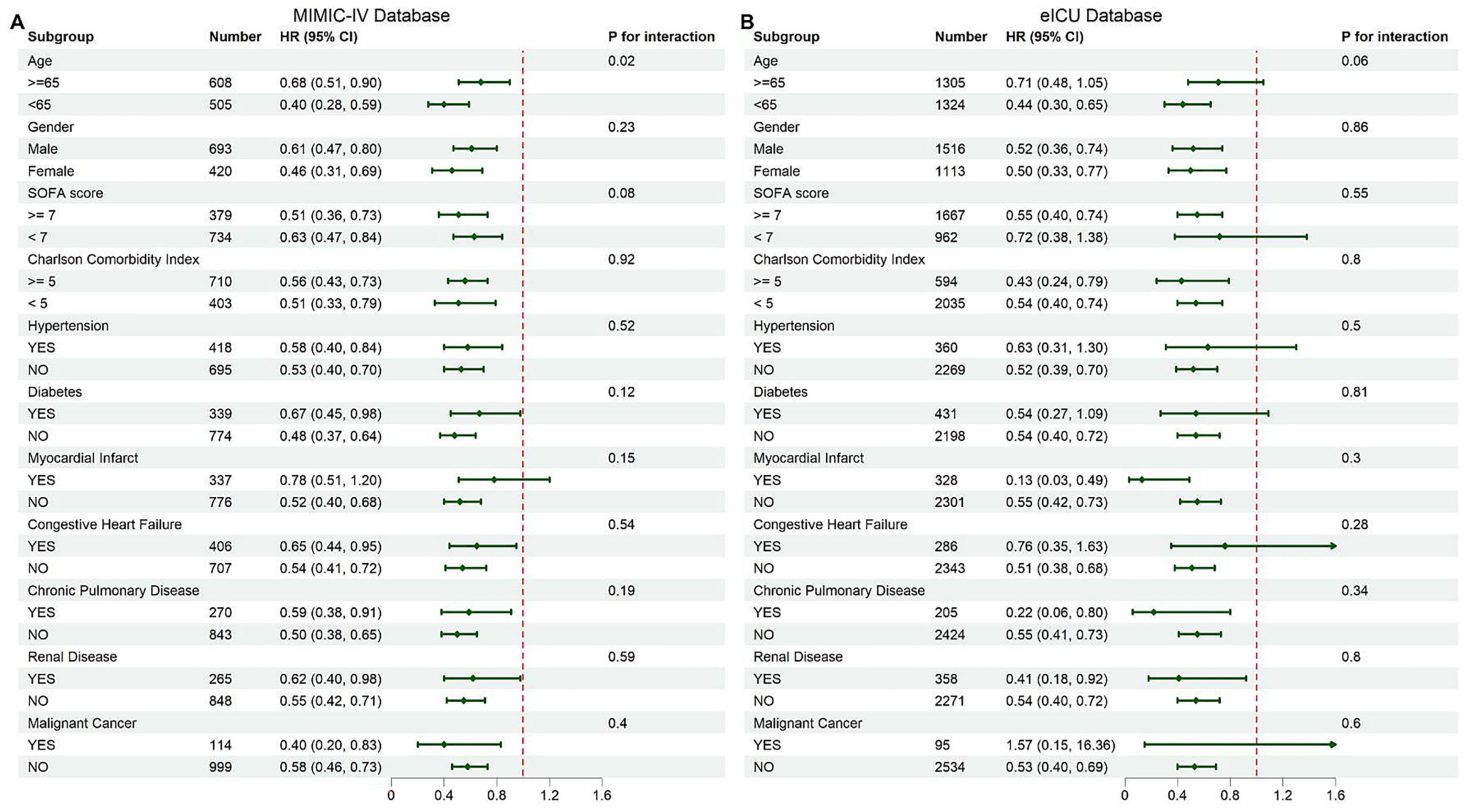
Subgroup analysis of in-hospital mortality: **(A)** MIMIC-IV, **(B)** eICU-CRD. Forest plots presenting hazard ratios for in-hospital mortality across predefined subgroups of cardiac arrest patients. **(A)** Results based on the MIMIC-IV cohort. **(B)** Results based on the eICU-CRD cohort. Subgroups include age, gender, SOFA score, Charlson Comorbidity Index, and comorbidities such as hypertension, diabetes, and chronic pulmonary disease. The dashed red line indicates a hazard ratio of 1 (no effect).

Subgroup analyses demonstrated a consistent association between multivitamin supplementation and reduced mortality across key clinical subgroups (Figure 5). In the MIMIC-IV cohort, the protective effect was observed across all predefined subgroups, with a stronger association in patients <65 years (*p* for interaction = 0.02). No significant interactions were found for other variables (*p* for interaction > 0.05). Similar trends were noted in the eICU-CRD validation cohort, where multivitamin use remained associated with lower mortality across subgroups, with no significant effect modification detected (*p* for interaction > 0.05). These findings suggest the survival benefit of multivitamin supplementation is consistent across clinically relevant populations.

### Duration of multivitamin supplementation and survival outcomes

**Figure 6:**
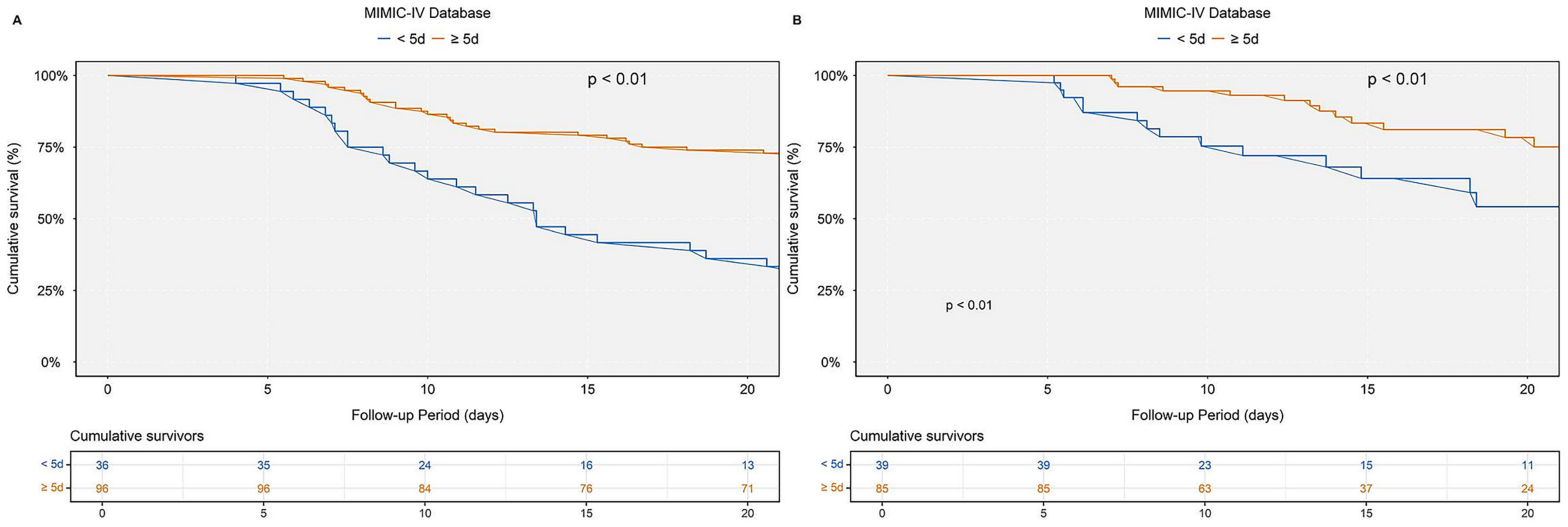
Kaplan–Meier survival curves for in-hospital mortality stratified by multivitamin use duration: **(A)** MIMIC-IV, **(B)** eICU-CRD. Kaplan–Meier survival curves comparing cumulative survival rates in patients receiving multivitamins for <5 days (blue) versus ≥5 days (orange). **(A)** Results based on the MIMIC-IV cardiac arrest cohort. **(B)** Results based on the eICU-CRD cardiac arrest cohort.

To assess the potential impact of multivitamin supplementation duration on survival, a stratified analysis was conducted among patients hospitalized for ≥5 days, categorizing them into two groups: those receiving multivitamins for <5 days and those for ≥5 days. Kaplan–Meier survival curves revealed a significantly higher cumulative survival probability in patients with ≥5 days of multivitamin use compared to those with <5 days (log-rank test, *p* < 0.01). This trend was consistently observed in both the MIMIC-IV and eICU-CRD cardiac arrest cohorts, as depicted in Figure 6.

## Discussion

This multicenter retrospective study, utilizing the MIMIC-IV cohort as the discovery set and the eICU-CRD cohort as the validation set, identified an association between multivitamin supplementation and reduced ICU, in-hospital, and 28-day mortality in critically ill cardiac arrest patients. These findings remained consistent after PSM and IPTW adjustment, supporting a potential beneficial relationship of multivitamin use in this population. Subgroup analyses suggested a more pronounced association in patients aged <65 years, with no significant interactions across other clinical subgroups. Furthermore, extended multivitamin supplementation (≥5 days) was associated with lower mortality, compatible with a potential duration-dependent pattern. Collectively, these results are consistent with a potential adjunctive role for multivitamin use in post-cardiac arrest care.

Post-cardiac arrest outcomes are strongly influenced by early post-return of spontaneous circulation (ROSC) care, including temperature management, hemodynamic optimization, targeted oxygenation and ventilation, and seizure surveillance and treatment (13). In line with these domains, our models adjusted for targeted temperature management, mechanical ventilation, vasopressor use, and renal replacement therapy, and the association estimates were similar after these adjustments. Contemporary randomized evidence shows no advantage of hypothermia at 33°C compared with active normothermia with fever prevention after out-of-hospital cardiac arrest (14). Similarly, targeted mild hypercapnia did not improve outcomes compared with normocapnia in comatose survivors (15). Restrictive versus liberal oxygenation strategies and higher versus lower mean arterial pressure targets also showed no significant differences in clinical outcomes (16). In this context of evolving and often neutral standard targets, exploration of adjunctive approaches remains warranted, and multivitamin supplementation represents one such candidate for study.

Numerous studies have explored the potential health benefits of multivitamin supplementation, yet definitive conclusions remain elusive. Most evidence concerns primary prevention in generally healthy populations rather than critical illness. A large-scale prospective study suggested that long-term multivitamin use (≥20 years) was associated with a reduced risk of major cardiovascular events, though no significant associations were found with myocardial infarction, stroke, or cardiovascular mortality (17). Another prospective analysis reported a modest association with lower cardiovascular disease risk, appearing stronger in participants younger than 60 years and in smokers (18). This aligns with our observation of a stronger association in patients younger than 65 years in the MIMIC-IV cohort. These results suggest that multivitamin supplementation may have enhanced benefits in younger patients, though further research is needed to confirm these observations. Despite these findings, the U. S. Preventive Services Task Force does not recommend routine multivitamin use for cardiovascular prevention because evidence remains insufficient to define a clear risk–benefit profile (19). By contrast, critically ill patients, particularly after cardiac arrest, often exhibit micronutrient deficits due to limited intake, increased metabolic demand, and therapy-related losses; restrictive feeding in the acute phase may worsen oxidative stress and immune dysregulation (20, 21). Preclinical data provide biological plausibility: in a rabbit ischemia–reperfusion model, antioxidant vitamins given before ischemia but not before reperfusion were associated with reduced tissue injury (22), and in an obesity-related model, multivitamin supplementation improved metabolic indices while reducing oxidative and inflammatory responses (23). These findings support biologic plausibility but do not establish clinical efficacy, warranting further investigation in post-cardiac arrest populations.

Cardiac arrest and resuscitation trigger ischemia–reperfusion injury, characterized by oxidative stress, systemic inflammation, mitochondrial dysfunction, and endothelial damage (24). ATP depletion and metabolic disruption during ischemia are followed by ROS production, inflammatory cytokine release, and immune activation upon reperfusion, exacerbating multi-organ dysfunction (25). Within this pathophysiology, multivitamin supplementation may modulate oxidative stress, inflammation, and cellular metabolism. B-vitamins support mitochondrial function and redox homeostasis, and thiamine may preserve pyruvate dehydrogenase activity, thereby contributing to these effects (26). B-complex preparations, including combinations with alpha-lipoic acid, demonstrate antioxidative effects in ischemia–reperfusion models (27). Vitamin B12 may limit ROS-mediated injury via SIRT3/AMPK, and vitamin B6/pyridoxal-5′-phosphate may attenuate calcium overload and ischemic dysfunction (28). Vitamin B6/pyridoxal-5′-phosphate may also protect against ischemic cardiac dysfunction (29). Vitamin C is central to redox and inflammatory control in this setting and has been associated with mitigation of myocardial and cerebral dysfunction after resuscitation (8, 30). Serum vitamin C levels are markedly depleted in post-arrest patients (31). In addition, experimental work indicates effects on oxidative stress, calcium handling, and mitochondrial integrity (32). Moreover, additional studies suggest involvement of PI3K-Akt and mitoKATP pathways (33). Vitamin D, recognized for cardioprotective effects, may augment multivitamin-related protection after cardiac arrest, with deficiency associated with higher risks of sudden cardiac arrest and mortality (34, 35). Observational and mechanistic data further suggest neuroprotection through Nrf2/HO-1 activation (36). Protective effects against ischemia-induced kidney injury have also been reported (37). Cardiac models describe reduced infarct size and inflammation with supplementation (38). In complementary fashion, vitamin E limits membrane lipid peroxidation and mitigates kidney injury in ischemia models (39). Neuroprotective effects have been described in cerebral ischemia when combined with beta-blockers (40). In cardiac models, *α*-tocopherol preserves myocardial function and reduces neutrophil-mediated inflammation (41). Beyond single-nutrient effects, multivitamin regimens in critical illness have been associated with reduced inflammation; in ICU-admitted COVID-19 patients, combined vitamins A, B, C, D, and E were linked to lower disease severity and systemic inflammation (42). Collectively, these observations are consistent with a potential advantage of multivitamin over single-vitamin supplementation in mitigating post-arrest ischemia–reperfusion-related inflammation and oxidative stress.

Balanced against potential benefits, multinutrient regimens may introduce unnecessary components and make safety prediction difficult in heterogeneous ICU populations. Excess intake of fat-soluble vitamins raises toxicity concerns, including vitamin D–related hypercalcemia and end-organ injury (43). Additionally, vitamin A toxicity has also been well documented in clinical practice and reviews (44). Moreover, high-dose vitamin E has been linked to higher all-cause mortality, and it has also been associated with an increased risk of haemorrhagic stroke (45, 46). Interactions with concurrent therapies matter; vitamin K can antagonize warfarin and other vitamin K antagonists (47). Finally, signals of harm have also been observed with specific regimens, including increased death or persistent organ dysfunction with intravenous vitamin C in ICU sepsis (48). Overall, these considerations underscore the need to define composition, dosing, timing, and co-therapies in future work, while situating our patient-level findings within a biologically plausible, hypothesis-generating framework.

This study has several limitations that should be acknowledged. First, as this is a retrospective observational study, causal inference cannot be drawn, and residual confounding from unmeasured variables may remain despite adjustment with PSM and IPTW. Second, the databases do not reliably record arrest location or a harmonized arrest or ROSC time, so we could not classify out-of-hospital versus in-hospital events or align multivitamin use with a specific post-ROSC day. Formulation and dose are inconsistently encoded, preventing dose–response analyses and introducing exposure heterogeneity. Third, neurological functional outcome data were insufficient, precluding analysis; we therefore focused on all-cause mortality and recommend inclusion of standardized neurological endpoints in future work. Fourth, baseline serum vitamin concentrations were unavailable, which precluded assessment of whether pre-existing deficiencies modified risk. Future large-scale, multicenter randomized controlled trials are needed to confirm these findings and establish whether multivitamin supplementation directly improves survival outcomes in post-cardiac arrest patients.

## Conclusion

In conclusion, we observed statistically significant associations between multivitamin supplementation and lower ICU, in-hospital, and 28-day mortality among post-cardiac arrest patients. Estimates were consistent after PSM and IPTW, and a longer supplementation duration (≥5 days) was also associated with lower mortality. Taken together, the observed associations support consideration of multivitamin use as a promising adjunctive therapy in post-cardiac arrest care.

## Data Availability

The raw data supporting the conclusions of this article will be made available by the authors, without undue reservation.
