# Supplementary material for: Multivitamin supplementation as a potential adjunctive therapy in post-cardiac arrest: insights from a multicenter retrospective analysis of MIMIC-IV and eICU-CRD
Source: Front Nutr. 2025 Sep 23;12:1602372. doi: 10.3389/fnut.2025.1602372 (PMC12505495; doi:10.3389/fnut.2025.1602372)
Supplement: Supplementary file 1 [file Data_Sheet_1.docx]

**Multivitamin Supplementation as a Potential Adjunctive Therapy in Post-Cardiac Arrest: Insights from a Multicenter Retrospective Analysis of MIMIC-IV and eICU-CRD**

Jiahao Shen ^#1^, Danjun Wang ^#2^, Wenxuan Zhao ^#3,4^, Jun Que ^*5^, Junwei Qian ^*6^, Xiaoyun Zhang^*7^

1 Trauma Center, Tongji Hospital, School of Medicine, Tongji University, Shanghai, 200065, PR China.

2 Department of Anesthesiology, Shanghai Geriatric Medical Center, No. 2560, Chunshen Road, Minhang District, Shanghai, 201104, PR China.

3 Tianjin Medical University, Tianjin, 300041, PR China.

4 Department of neurosurgery, Jiangnan University Medical Center, Jiangnan university, Wuxi, 214122, PR China.

5 Department of Thoracic Surgery, the First Affiliated Hospital with Nanjing Medical University, PR China.

6 Department of Emergency Medicine, Huashan Hospital, Fudan University, 12 Wulumuqi Zhong Road, Shanghai, 200040, PR China.

7 Department of Rheumatology, Huashan Hospital, Fudan University, Shanghai, 200040, PR China.

* Correspondence to: Xiaoyun Zhang, Department of Rheumatology, Huashan Hospital, Fudan University, Shanghai, 200040, China. E-mail: [doc_zxy@126.com](mailto:doc_zxy@126.com). Junwei Qian, Department of Emergency Medicine, Huashan Hospital, Fudan University, 12 Wulumuqi Zhong Road, Shanghai, 200040, China. E-mail: [qjw199708@163.com](mailto:qjw199708@163.com). Jun Que, Department of Thoracic Surgery, the First Affiliated Hospital with Nanjing Medical University, E-mail: [2606633062@qq.com](mailto:2606633062@qq.com).

# These authors are contributed equally to this work.

**Supplemental Digital Content**

[**Supplementary Table** 3](#_Toc204379396)

[Supplementary Table 1 Multivitamin Definition 3](#_Toc204379397)

[Supplementary Table 2 Baseline Characteristics of Original Patients in the eICU-CRD Cardiac Arrest Cohort 4](#_Toc204379398)

[Supplementary Table 3 Hazard Ratios for Multivitamin Use on Mortality in eICU-CRD Cardiac Arrest Cohort 6](#_Toc204379399)

[Supplementary Table 4 Baseline Characteristics of Patients After PSM in the MIMIC-IV Cardiac Arrest Cohort 7](#_Toc204379400)

[Supplementary Table 5 Baseline Characteristics of Patients After PSM in the eICU-CRD Cardiac Arrest Cohort 9](#_Toc204379401)

[**Supplementary Figure** 11](#_Toc204379402)

[Supplementary Figure 1 Distribution of Missing Values in Cardiac Arrest Cohorts: (A) MIMIC-IV, (B) eICU-CRD 11](#_Toc204379403)

[Supplementary Figure 3 Survival Analysis by Multivitamin Use in eICU-CRD Cardiac Arrest Cohort 13](#_Toc204379404)

[Supplementary Figure 4 Standardized Mean Differences of Covariates Before and After PSM and IPTW: (A) MIMIC-IV, (B) eICU-CRD 14](#_Toc204379405)

[Supplementary Figure 5 Kernel Density of Propensity Scores Before and After PSM and IPTW: (A) MIMIC-IV, (B) eICU-CRD 15](#_Toc204379406)

[Supplementary Figure 6 Survival Analysis After PSM and IPTW for Multivitamin Use in the eICU-CRD Cardiac Arrest Cohort 16](#_Toc204379407)

# **Supplementary Table**

## **Supplementary Table 1 Multivitamin Definition**

| **Database** | **Definition and Selection Logic** | **Drug Codes** | **Drug Names** |
| --- | --- | --- | --- |
| **eICU** | Records were included if the drug name matched “multivitamin” (case-insensitive) and did not contain the term “prenatal”. | **drughiclseqno:** 1086, 1095, 1096, 1097, 1098, 1099, 21996, 26691, 35344, 36510, 36919, 38448 | ‘MVI’, ‘MULTIVITAMIN’, ‘MULTIVITAMINS’, ‘INFUVITE IV INJ 10ML’, ‘VITAMINS/MINERALS PO TABS’, ‘MVI, ADULT NO.4 WITH VIT K 3300 UNIT-150 MCG/10 ML IV 10 ML VIAL’ |
| **MIMIC-IV** |  | **ndc:** 00904549261, 00005434462, 00245008201, 00245009101, 00904053061, 00904504260, 00904549213, 00904549261, 16500008806, 50383068304, 54643564901, 66591018442 | ‘multivitamin’, ‘Multivitamins’,  ‘Multivitamin IV’,  ‘Multivitamins W/minerals’, ‘Multivitamins W/minerals Liquid’, ‘Multivitamins W/minerals Chewable’ |

## **Supplementary Table 2 Baseline Characteristics of Original Patients in the eICU-CRD Cardiac Arrest Cohort**

| **Variables** | **All**  **(n = 2629)** | **Multivitamin**  **(n = 174)** | **No Multivitamin**  **(n = 2455)** | ***p-*value** |
| --- | --- | --- | --- | --- |
| **Age, years** | 64 (53, 74) | 61 (49, 72) | 65 (54, 74) | < 0.01 |
| **Male, %** | 1516 (57.7%) | 110 (63.2%) | 1406 (57.3%) | 0.15 |
| **CCI score** | 4 (2, 4) | 3 (2, 4) | 4 (2, 4) | 0.04 |
| **SOFA score** | 8 (5, 11) | 9 (6, 12) | 8 (5, 11) | 0.01 |
| **Race, n (%)** |  |  |  | 0.39 |
| Whtie | 1922 (73.1%) | 136 (78.2%) | 1786 (72.7%) |  |
| Black | 376 (14.3%) | 18 (10.3%) | 358 (14.6%) |  |
| Asian | 51 (1.9%) | 2 (1.1%) | 49 (2%) |  |
| Other | 280 (10.7%) | 18 (10.3%) | 262 (10.7%) |  |
| **Comorbidities, n (%)** |  |  |  |  |
| Hypertension | 360 (13.7%) | 25 (14.4%) | 335 (13.6%) | 0.88 |
| Diabetes mellitus | 431 (16.4%) | 27 (15.5%) | 404 (16.5%) | 0.83 |
| Myocardial infarct | 328 (12.5%) | 19 (10.9%) | 309 (12.6%) | 0.60 |
| Congestive heart failure | 286 (10.9%) | 23 (13.2%) | 263 (10.7%) | 0.37 |
| Chronic pulmonary disease | 205 (7.8%) | 11 (6.3%) | 194 (7.9%) | 0.55 |
| Renal disease | 358 (13.6%) | 24 (13.8%) | 334 (13.6%) | 1.00 |
| Malignant cancer | 95 (3.6%) | 4 (2.3%) | 91 (3.7%) | 0.45 |
| **Laboratory tests** |  |  |  |  |
| Hemoglobin, g/dL | 11.6 (9.6, 13.5) | 11.1 (9.1, 13.2) | 11.6 (9.6, 13.6) | 0.04 |
| WBC, 10^9^ /L | 13.6 (9.5, 19.1) | 13.7 (9.1, 18.6) | 13.6 (9.6, 19.1) | 0.49 |
| Platelet, 10^9^ /L | 198 (148, 253) | 188 (136, 258) | 199 (149, 253) | 0.11 |
| INR | 1.2 (1.1, 1.5) | 1.2 (1.1, 1.5) | 1.2 (1.1, 1.5) | 0.49 |
| Creatinine, mg/dl | 1.3 (0.9, 2.1) | 1.3 (0.8, 1.9) | 1.3 (0.9, 2.1) | 0.09 |
| Glucose, mg/dl | 168 (123, 237) | 154 (118, 211) | 169 (123, 238) | 0.02 |
| Bicarbonate, mmol/L | 22.0 (19.0, 25.0) | 22.0 (19.0, 26.0) | 22.0 (19.0, 25.0) | 0.97 |
| Lactate, mmol/L | 2.7 (1.5, 5.2) | 2.5 (1.5, 4.3) | 2.7 (1.5, 5.2) | 0.18 |
| pH | 7.3 (7.2, 7.4) | 7.3 (7.3, 7.4) | 7.3 (7.2, 7.4) | 0.04 |
| pO2, mmHg | 115.0 (76.0, 213.0) | 123.8 (79.2, 206.8) | 115.0 (76.0, 213.0) | 0.36 |
| **Vital signs** |  |  |  |  |
| HR, beats/minute | 89 (74, 106) | 96 (80, 111) | 88 (73, 105) | < 0.01 |
| RR, times/minute | 20 (16, 24) | 20 (17, 25) | 20 (16, 24) | 0.47 |
| MBP, mmHg | 83 (68, 98) | 84 (67, 98) | 83 (68, 98) | 0.91 |
| SpO2, % | 99 (95, 100) | 99 (95, 100) | 99 (95, 100) | 0.84 |
| Temperature, ℃ | 36.4 (35.6, 36.9) | 36.4 (35.9, 37.0) | 36.4 (35.6, 36.9) | 0.03 |
| **Therapies, n (%)** |  |  |  |  |
| Mechanical ventilation | 2047 (77.9%) | 137 (78.7%) | 1910 (77.8%) | 0.85 |
| Vasopressor | 1554 (59.1%) | 93 (53.4%) | 1461 (59.5%) | 0.14 |
| TTM | 511 (19.4%) | 21 (12.1%) | 490 (20.0%) | 0.01 |
| RRT | 271 (10.3%) | 16 (9.2%) | 255 (10.4%) | 0.71 |
| **Outcome** |  |  |  |  |
| ICU death | 990 (37.7%) | 48 (27.6%) | 942 (38.4%) | 0.01 |
| In-Hospital death | 1191 (45.3%) | 57 (32.8%) | 1134 (46.2%) | < 0.01 |
| 28-day death | 2538 (96.5%) | 157 (90.2%) | 2381 (97.0%) | < 0.01 |

CCI: Charlson Comorbidity Index, SOFA: Sequential Organ Failure Assessment, WBC: White Blood Cell count, INR: International Normalized Ratio, HR: Heart Rate, RR: Respiratory Rate, MBP: Mean Blood Pressure, SpO_2_: Peripheral Capillary Oxygen Saturation, TTM: Targeted Temperature Management, RRT: Renal Replacement Therapy, ICU: Intensive Care Unit.

## **Supplementary Table 3 Hazard Ratios for Multivitamin Use on Mortality in eICU-CRD Cardiac Arrest Cohort**

| **Outcomes** | **Model 1** | | **Model 2** | | **Model 3** | |
| --- | --- | --- | --- | --- | --- | --- |
|  | **HR (95% CIs)** | ***p-*value** | **HR (95% CIs)** | ***p*-value** | **HR (95% CIs)** | ***p*-value** |
| **Original population** | | | | | | |
| ICU mortality | | | | | | |
| Multivitamin | 0.52 (0.39, 0.70) | < 0.01 | 0.46 (0.34, 0.61) | < 0.01 | 0.56 (0.41, 0.75) | < 0.01 |
| In-Hospital death | | | | | | |
| Multivitamin | 0.50 (0.38, 0.65) | < 0.01 | 0.45 (0.34, 0.59) | < 0.01 | 0.53 (0.40, 0.70) | < 0.01 |
| 28-day death | | | | | | |
| Multivitamin | 0.57 (0.49, 0.68) | < 0.01 | 0.58 (0.49, 0.68) | < 0.01 | 0.61 (0.52, 0.72) | < 0.01 |
| **After PSM** | | | | | | |
| ICU mortality | | | | | | |
| Multivitamin | 0.48 (0.33, 0.71) | < 0.01 | 0.44 (0.30, 0.65) | < 0.01 | 0.43 (0.28, 0.65) | < 0.01 |
| In-Hospital death | | | | | | |
| Multivitamin | 0.50 (0.35, 0.72) | < 0.01 | 0.47 (0.33, 0.67) | < 0.01 | 0.45 (0.31, 0.65) | < 0.01 |
| 28-day death | | | | | | |
| Multivitamin | 0.57 (0.45, 0.71) | < 0.01 | 0.54 (0.43, 0.68) | < 0.01 | 0.50 (0.39, 0.64) | < 0.01 |
| **After IPTW** | | | | | | |
| ICU mortality | | | | | | |
| Multivitamin | 0.53 (0.39, 0.73) | < 0.01 | 0.54 (0.40, 0.73) | < 0.01 | 0.58 (0.44, 0.78) | < 0.01 |
| In-Hospital death | | | | | | |
| Multivitamin | 0.50 (0.38, 0.67) | < 0.01 | 0.51 (0.39, 0.67) | < 0.01 | 0.55 (0.42, 0.72) | < 0.01 |
| 28-day death | | | | | | |
| Multivitamin | 0.56 (0.47, 0.67) | < 0.01 | 0.58 (0.48, 0.69) | < 0.01 | 0.60 (0.51, 0.71) | < 0.01 |

HR, Hazard Ratio; CIs, Confidence Intervals; ICU, Intensive Care Unit; PSM, propensity score matching; IPTW: Inverse Probability of Treatment Weighting

Model 1: Adjusted for demographic characteristics.

Model 2: Adjusted for demographic characteristics, disease severity, and comorbidities.

Model 3: Adjusted for demographic characteristics, disease severity, comorbidities, laboratory markers, and treatment interventions.

## **Supplementary Table 4 Baseline Characteristics of Patients After PSM in the MIMIC-IV Cardiac Arrest Cohort**

| **Variables** | **All**  **(n = 430)** | **Multivitamin**  **(n = 215)** | **No Multivitamin**  **(n = 215)** | ***p-*value** |
| --- | --- | --- | --- | --- |
| **Age, years** | 66 (55, 77) | 67 (55, 77) | 66 (55, 76) | 0.73 |
| **Male, n (%)** | 291 (67.7%) | 142 (66.0%) | 149 (69.3%) | 0.54 |
| **CCI score** | 6 (4, 8) | 6 (4, 8) | 6 (4, 9) | 0.74 |
| **SOFA score** | 7 (5, 10) | 7 (5, 10) | 7 (4, 10) | 0.68 |
| **Race, n (%)** |  |  |  | 0.94 |
| Whtie | 230 (53.5%) | 112 (52.1%) | 118 (54.9%) |  |
| Black | 43 (10.0%) | 23 (10.7%) | 20 (9.3%) |  |
| Asian | 6 (1.4%) | 3 (1.4%) | 3 (1.4%) |  |
| Other | 151 (35.1%) | 77 (35.8%) | 74 (34.4%) |  |
| **Comorbidities, n (%)** |  |  |  |  |
| Hypertension | 135 (31.4%) | 68 (31.6%) | 67 (31.2%) | 1.00 |
| Diabetes mellitus | 127 (29.5%) | 67 (31.2%) | 60 (27.9%) | 0.53 |
| Myocardial infarct | 118 (27.4%) | 60 (27.9%) | 58 (27.0%) | 0.91 |
| Congestive heart failure | 183 (42.6%) | 92 (42.8%) | 91 (42.3%) | 1.00 |
| Chronic pulmonary disease | 109 (25.3%) | 54 (25.1%) | 55 (25.6%) | 1.00 |
| Renal disease | 118 (27.4%) | 60 (27.9%) | 58 (27.0%) | 0.91 |
| Malignant cancer | 54 (12.6%) | 25 (11.6%) | 29 (13.5%) | 0.66 |
| **Laboratory tests** |  |  |  |  |
| Hemoglobin, g/dL | 11.2 ± 2.7 | 11.2 ± 2.7 | 11.2 ± 2.7 | 0.96 |
| WBC, 10^9^ /L | 13.4 (9.2, 18.4) | 12.5 (9.1, 18.3) | 14.1 (9.4, 18.4) | 0.5 |
| Platelet, 10^9^ /L | 167 (125, 224) | 161 (125, 224) | 170 (124, 224) | 0.95 |
| INR | 1.4 (1.1, 1.8) | 1.4 (1.1, 1.8) | 1.4 (1.1, 1.8) | 0.83 |
| Creatinine, mg/dl | 1.3 (0.9, 1.9) | 1.3 (0.9, 1.9) | 1.3 (0.9, 2.0) | 0.34 |
| Glucose, mg/dl | 150 (113, 217) | 150 (110, 214) | 154 (116, 216) | 0.56 |
| Bicarbonate, mmol/L | 21 (18, 24) | 21 (18, 24) | 22 (18, 24) | 0.55 |
| Lactate, mmol/L | 2.6 (1.6, 4.6) | 2.6 (1.6, 4.6) | 2.7 (1.6, 4.6) | 0.46 |
| pH | 7.3 (7.2, 7.4) | 7.3 (7.2, 7.4) | 7.3 (7.2, 7.4) | 0.22 |
| pO2, mmHg | 90 (49, 200) | 85 (47, 200) | 97 (50, 196) | 0.53 |
| **Vital signs** |  |  |  |  |
| HR, beats/minute | 81 (70, 955) | 82 (71, 94) | 80 (69, 95) | 0.55 |
| RR, times/minute | 19 (17, 23) | 19 (17, 24) | 19 (17, 23) | 0.52 |
| SpO2, % | 97 (96, 99) | 97 (96, 99) | 97 (96, 99) | 0.52 |
| **Therapies, n (%)** |  |  |  |  |
| Mechanical ventilation | 372 (86.5%) | 188 (87.4%) | 184 (85.6%) | 0.67 |
| Vasopressor | 349 (81.2%) | 172 (80.0%) | 177 (82.3%) | 0.62 |
| TTM | 181 (42.1%) | 94 (43.7%) | 87 (40.5%) | 0.56 |
| RRT | 86 (20.0%) | 43 (20.0%) | 43 (20.0%) | 1 |
| **Outcome, n (%)** |  |  |  |  |
| ICU death | 204 (47.4%) | 87 (40.5%) | 117 (54.4%) | < 0.01 |
| In-Hospital death | 232 (54.0%) | 102 (47.4%) | 130 (60.5%) | < 0.01 |
| 28-day death | 230 (53.5%) | 99 (46.0%) | 131 (60.9%) | < 0.01 |

## **Supplementary Table 5 Baseline Characteristics of Patients After PSM in the eICU-CRD Cardiac Arrest Cohort**

| **Variables** | **All**  **(n = 346)** | **Multivitamin**  **(n = 173)** | **No Multivitamin**  **(n = 173)** | ***p-*value** |
| --- | --- | --- | --- | --- |
| **Age, years** | 62 (51, 72) | 61 (49, 72) | 62 (52, 73) | 0.26 |
| **Male, n (%)** | 223 (64.5%) | 110 (63.6%) | 113 (65.3%) | 0.82 |
| **CCI score** | 3 (2, 4) | 3 (2, 4) | 4 (2, 4) | 0.92 |
| **SOFA score** | 9 (6, 12) | 9 (6, 12) | 9 (5, 12) | 0.68 |
| **Race, n (%)** |  |  |  | 0.89 |
| Whtie | 269(77.7%) | 135(78%) | 134(77.5%) |  |
| Black | 34(9.8%) | 18(10.4%) | 16(9.2%) |  |
| Asian | 6(1.7%) | 2(1.2%) | 4(2.3%) |  |
| Other | 37(10.7%) | 18(10.4%) | 19(11%) |  |
| **Comorbidities, n (%)** |  |  |  |  |
| Hypertension | 50 (14.5%) | 25 (14.5%) | 25 (14.5%) | 1.00 |
| Diabetes mellitus | 54 (15.6%) | 27 (15.6%) | 27 (15.6%) | 1.00 |
| Myocardial infarct | 34 (9.8%) | 19 (11.0%) | 15 (8.7%) | 0.59 |
| Congestive heart failure | 46 (13.3%) | 23 (13.3%) | 23 (13.3%) | 1.00 |
| Chronic pulmonary disease | 20 (5.8%) | 11 (6.4%) | 9 (5.2%) | 0.82 |
| Renal disease | 45 (13.0%) | 24 (13.9%) | 21 (12.1%) | 0.75 |
| Malignant cancer | 7 (2.0%) | 4 (2.3%) | 3 (1.7%) | 1.00 |
| **Laboratory tests** |  |  |  |  |
| Hemoglobin, g/dL | 11.1 (9.3, 13.0) | 11.1 (9.2, 13.1) | 11.0 (9.3, 12.9) | 0.89 |
| WBC, 10^9^ /L | 13.0 (8.8, 18.6) | 13.7 (9.0, 18.6) | 12.6 (8.7, 18.5) | 0.77 |
| Platelet, 10^9^ /L | 186 (133, 251) | 186 (134, 258) | 185 (131, 244) | 0.89 |
| INR | 1.2 (1.1, 1.5) | 1.2 (1.1, 1.5) | 1.2 (1.1, 1.4) | 0.37 |
| Creatinine, mg/dl | 1.2 (0.9, 1.9) | 1.2 (0.8, 1.9) | 1.1 (0.9, 1.9) | 0.97 |
| Glucose, mg/dl | 152 (116, 212) | 153 (116, 210) | 150 (116, 214) | 0.94 |
| Bicarbonate, mmol/L | 22.3 ± 5.5 | 22.2 ± 5.8 | 22.4 ± 5.2 | 0.76 |
| Lactate, mmol/L | 2.5 (1.5, 4.3) | 2.6 (1.5, 4.4) | 2.4 (1.4, 4.2) | 0.30 |
| pH | 7.3 (7.3, 7.4) | 7.3 (7.3, 7.4) | 7.3 (7.3, 7.4) | 0.96 |
| pO2, mmHg | 119.1 (77.0, 217.7) | 127.0 (79.0, 208.0) | 118.0 (74.0, 241.0) | 0.96 |
| **Vital signs** |  |  |  |  |
| HR, beats/minute | 97 (81, 113) | 97 (82, 111) | 97 (81, 115) | 0.70 |
| RR, times/minute | 20 (17, 26) | 20 (17, 26) | 21 (18, 26) | 0.22 |
| SpO2, % | 99 (94, 100) | 99 (94, 100) | 99 (94, 100) | 0.79 |
| **Therapies, n (%)** |  |  |  |  |
| Mechanical ventilation | 281 (81.2%) | 137 (79.2%) | 144 (83.2%) | 0.41 |
| Vasopressor | 177 (51.2%) | 92 (53.2%) | 85 (49.1%) | 0.52 |
| TTM | 39 (11.3%) | 21 (12.1%) | 18 (10.4%) | 0.73 |
| RRT | 32 (9.2%) | 16 (9.2%) | 16 (9.2%) | 1.00 |
| **Outcome, n (%)** |  |  |  |  |
| ICU death | 117 (33.8%) | 48 (27.7%) | 69 (39.9%) | 0.02 |
| In-Hospital death | 134 (38.7%) | 57 (32.9%) | 77 (44.5%) | 0.04 |
| 28-day death | 324 (93.6%) | 156 (90.2%) | 168 (97.1%) | 0.02 |

# **Supplementary Figure**


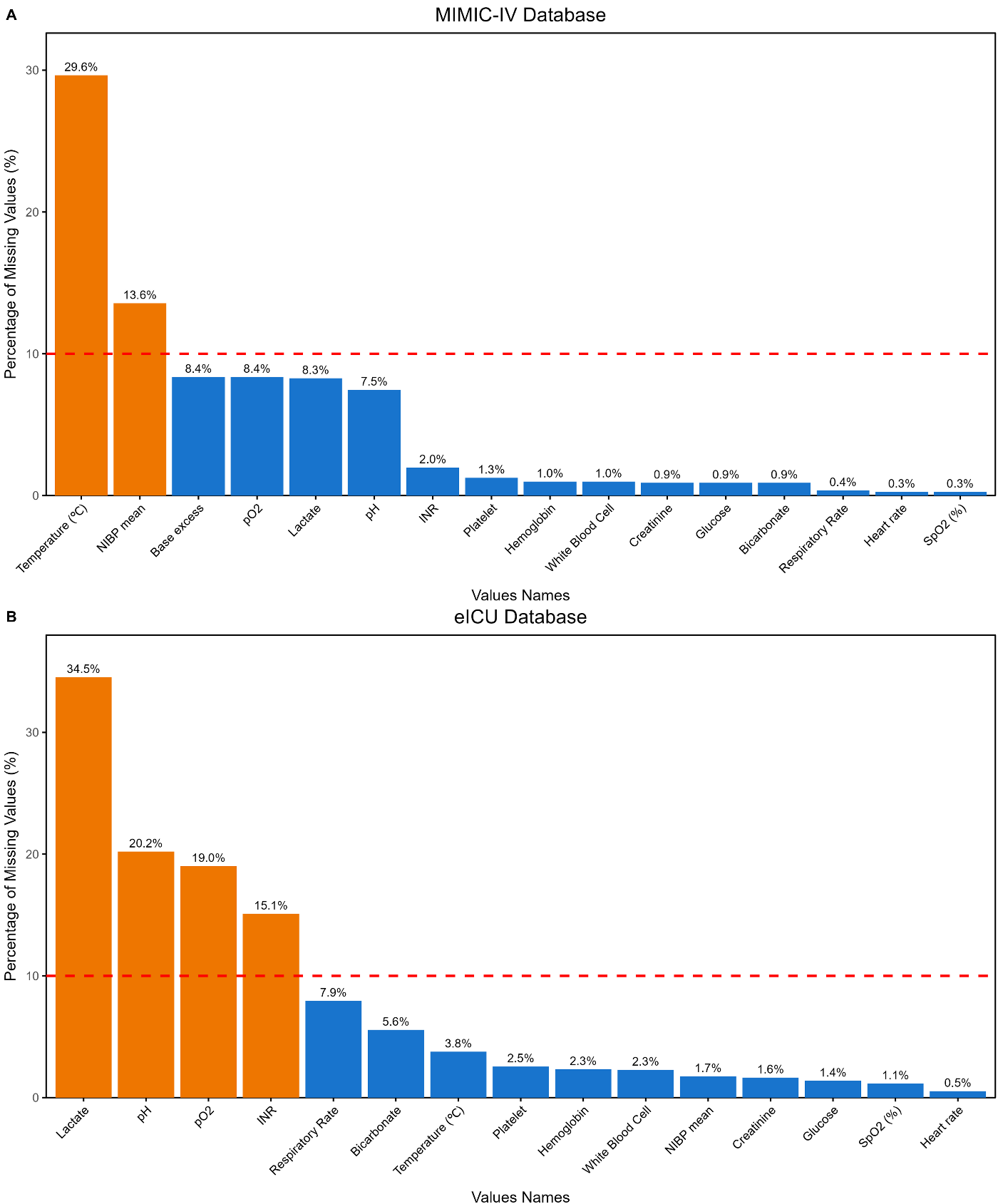


## **Supplementary Figure 1 Distribution of Missing Values in Cardiac Arrest Cohorts: (A) MIMIC-IV, (B) eICU-CRD**

Variables with missing values exceeding 10% (denoted by the red dashed line) will be excluded from subsequent analyses.


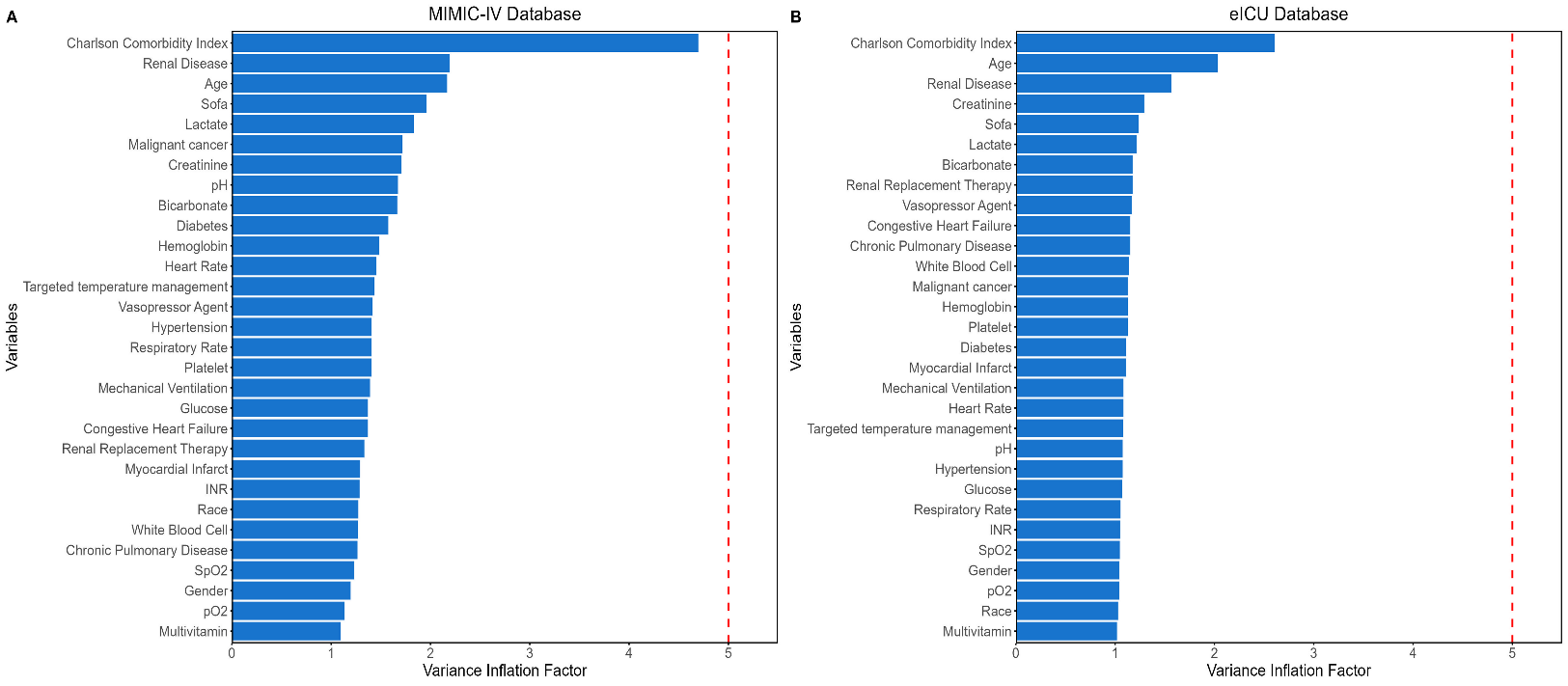

**Supplementary Figure 2 Variance Inflation Factors in Fully Adjusted Cox Regression Model: (A) MIMIC-IV, (B) eICU-CRD**

(A) VIF values for the MIMIC-IV cardiac arrest cohort. (B) VIF values for the eICU-CRD cardiac arrest cohort. The red dashed line represents the VIF threshold of 5, indicating potential multicollinearity.


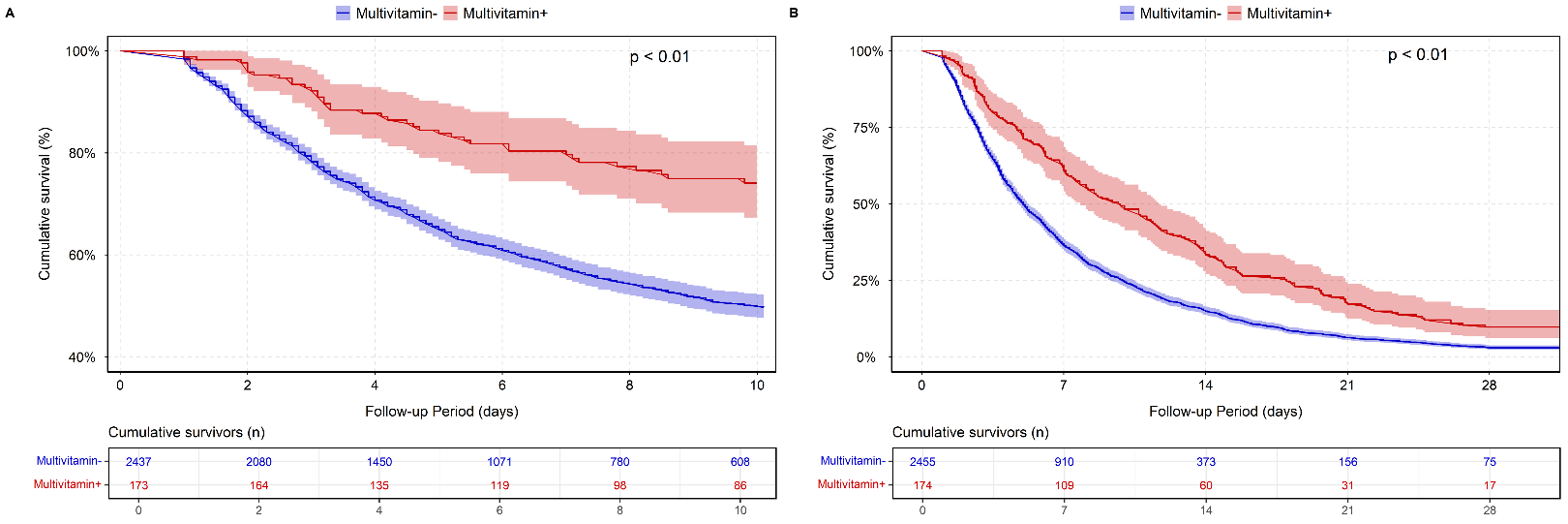


## **Supplementary Figure 3 Survival Analysis by Multivitamin Use in eICU-CRD Cardiac Arrest Cohort**

**(A) In-Hospital Survival, (B) 28-Day Survival**


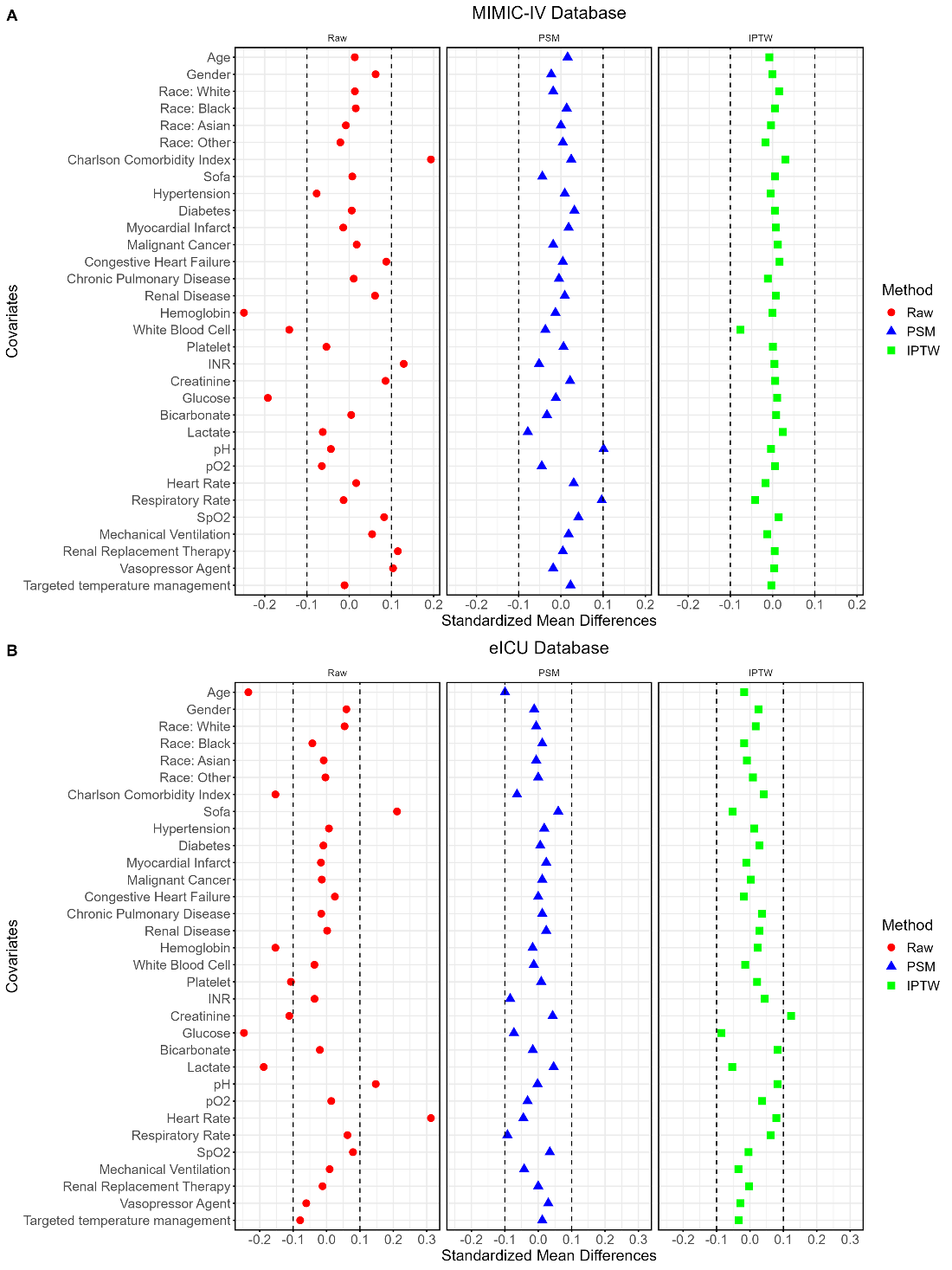


## **Supplementary Figure 4 Standardized Mean Differences of Covariates Before and After PSM and IPTW: (A) MIMIC-IV, (B) eICU-CRD**

(A) Covariate balance in the MIMIC-IV database. (B) Covariate balance in the eICU-CRD database. Each panel represents the covariate balance under three conditions: Raw (unadjusted, red circles), PSM (blue triangles), and IPTW (green squares). The dashed vertical lines at ±0.1 represent the threshold for acceptable balance, with values closer to zero indicating better covariate balance.

**
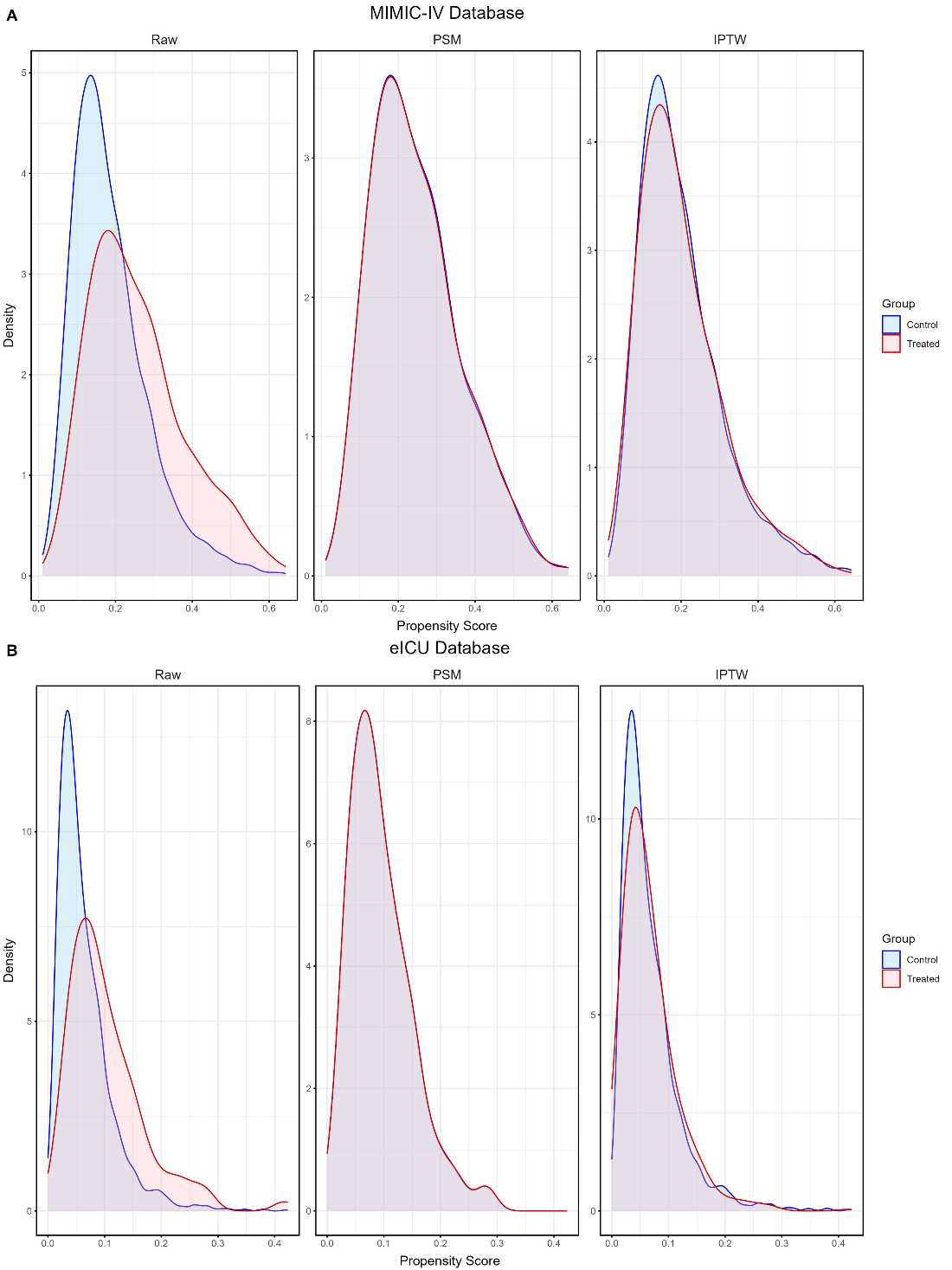
**

## **Supplementary Figure 5 Kernel Density of Propensity Scores Before and After PSM and IPTW: (A) MIMIC-IV, (B) eICU-CRD**

Kernel density distributions of propensity scores for the treatment group (red) and control group (blue) are shown. The distributions are displayed for raw data (left), after propensity score matching (PSM, middle), and after inverse probability of treatment weighting (IPTW, right). Panel A corresponds to the MIMIC-IV cardiac arrest cohort, and Panel B corresponds to the eICU-CRD cardiac arrest cohort.


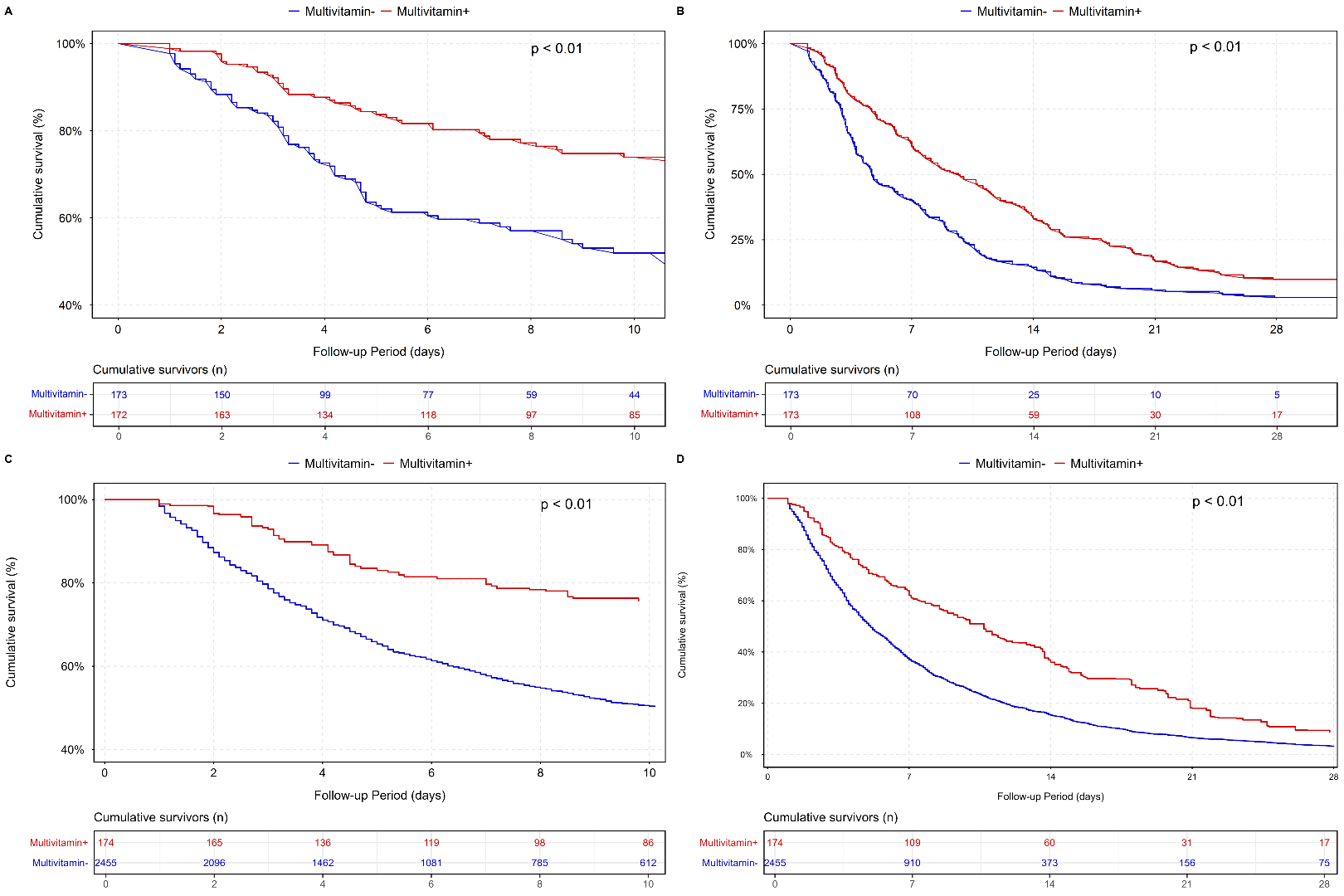


## **Supplementary Figure 6 Survival Analysis After PSM and IPTW for Multivitamin Use in the eICU-CRD Cardiac Arrest Cohort**

Panel A shows the Kaplan-Meier survival curve for in-hospital mortality after PSM. Panel B displays the same comparison over a 28-day follow-up period after PSM. Panel C presents the in-hospital survival curve after IPTW, and Panel D shows the 28-day survival curve after IPTW.
